# *catena*-Poly[[[tetra­aqua­lanthanum(III)]-di-μ-isonicotinato-κ^4^ *O*:*O*′] chloride]

**DOI:** 10.1107/S1600536812027778

**Published:** 2012-06-27

**Authors:** Jin-He Zhao

**Affiliations:** a Department of Chemical and Life Science, Baise University, Baise 533000, People’s Republic of China

## Abstract

In the title compound, {[La(C_6_H_4_NO_2_)_2_(H_2_O)_4_]Cl}_*n*_, the La^III^ atom lies on a twofold rotation axis and is eight-coordinated by four O atoms from four isonicotinate ligands and four water mol­ecules in a distorted square-anti­prismatic coodination environment. Adjacent La^III^ atoms are bridged by two carboxyl­ate groups from two isonicotinate ligands, forming an extended chain along [001]. These chains are linked through O—H⋯N hydrogen bonds into a three-dimensional network with channels in which the chloride anions form O—H⋯Cl hydrogen bonds. Intra­chain O—H⋯O hydrogen bonds and π–π inter­actions [centroid–centroid distance = 3.908 (2) Å] are also observed.

## Related literature

For lanthanide complexes with nicotinic acid, isonicotinic acid and isonicotinic acid *N*-oxide ligands, see: Cai *et al.* (2003 ▶); Chen & Fukuzumi (2009 ▶); Cui *et al.* (1999 ▶); Kay *et al.* (1972 ▶); Ma *et al.* (1996 ▶, 1999 ▶); Mao *et al.* (1998 ▶); Starynowicz (1991 ▶, 1993 ▶); Wu *et al.* (2008 ▶); Zeng *et al.* (2000 ▶); Zhang *et al.* (1999 ▶).
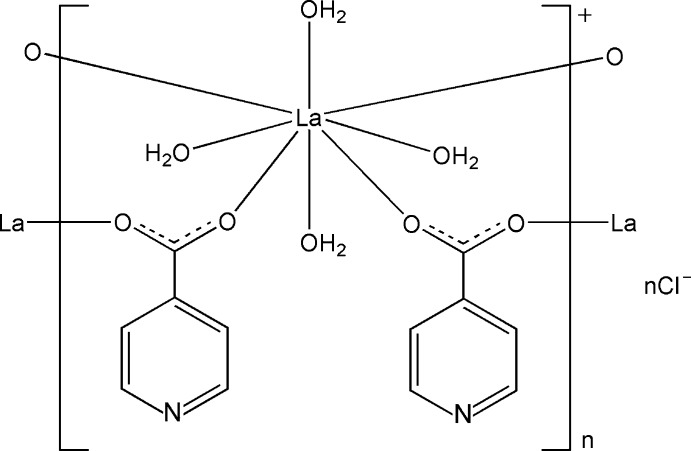


## Experimental

### 

#### Crystal data

[La(C_6_H_4_NO_2_)_2_(H_2_O)_4_]Cl
*M* *_r_* = 490.63
Orthorhombic, 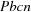

*a* = 8.987 (3) Å
*b* = 19.769 (3) Å
*c* = 10.305 (3) Å
*V* = 1830.8 (9) Å^3^
*Z* = 4
Mo *K*α radiation
μ = 2.52 mm^−1^
*T* = 296 K
0.36 × 0.34 × 0.32 mm

#### Data collection

Bruker SMART 1000 CCD diffractometer
Absorption correction: multi-scan (*SADABS*; Sheldrick, 1996 ▶) *T*_min_ = 0.464, *T*_max_ = 0.500
9336 measured reflections
1652 independent reflections
1442 reflections with *I* > 2σ(*I*)
*R*_int_ = 0.029

#### Refinement

*R*[*F* ^2^ > 2σ(*F* ^2^)] = 0.025
*wR*(*F* ^2^) = 0.058
*S* = 1.07
1652 reflections
110 parameters
H-atom parameters constrained
Δρ_max_ = 0.46 e Å^−3^
Δρ_min_ = −0.83 e Å^−3^

### 

Data collection: *SMART* (Bruker, 2007 ▶); cell refinement: *SAINT* (Bruker, 2007 ▶); data reduction: *SAINT*; program(s) used to solve structure: *SHELXS97* (Sheldrick, 2008 ▶); program(s) used to refine structure: *SHELXL97* (Sheldrick, 2008 ▶); molecular graphics: *DIAMOND* (Brandenburg, 1999 ▶); software used to prepare material for publication: *SHELXTL* (Sheldrick, 2008 ▶).

## Supplementary Material

Crystal structure: contains datablock(s) I, global. DOI: 10.1107/S1600536812027778/hy2530sup1.cif

Structure factors: contains datablock(s) I. DOI: 10.1107/S1600536812027778/hy2530Isup2.hkl

Additional supplementary materials:  crystallographic information; 3D view; checkCIF report

## Figures and Tables

**Table 1:** Hydrogen-bond geometry (Å, °)

*D*—H⋯*A*	*D*—H	H⋯*A*	*D*⋯*A*	*D*—H⋯*A*
O3—H3*A*⋯N1^i^	0.85	1.85	2.699 (4)	175
O3—H3*B*⋯Cl1^ii^	0.85	2.36	3.212 (2)	175
O4—H4*C*⋯O3^iii^	0.85	2.01	2.860 (3)	180
O4—H4*D*⋯Cl1	0.85	2.17	3.024 (3)	180

Symmetry codes: (i) 
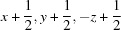
; (ii) 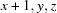; (iii) 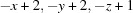.

**Fig. 1.**
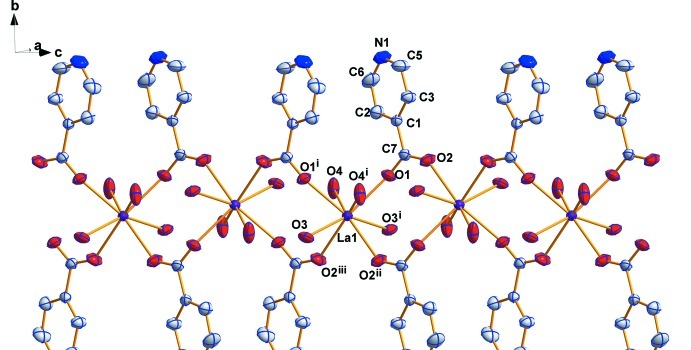
Part of the one-dimensional cationic chain of the title compound (Cl anions are not shown). Displacement ellipsoids are shown at the 30% probability level. [Symmetry codes: (i) 2-x, y, 1/2-z; (ii) 2-x, 2-y, -z; (iii) x, 2-y, 1/2+z.]

**Fig. 2.**
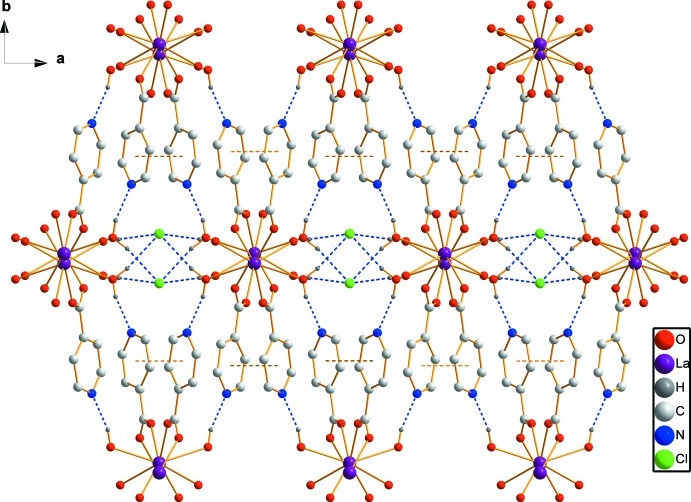
Packing diagram of the title compound. Yellow dashed lines represent π–π interactions and green dashed lines represent hydrogen bonds.

## supplementary crystallographic information

### Comment

Much attention has been devoted to the research on lanthanide metal polynuclear compounds because of their magnetic and luminescent properties. Most of these types of compounds were synthesized by the reaction of rare-earth metal ions with bi- or multi-dentate ligands such as nicotinic acid (Kay *et al.*, 1972; Ma, Hu *et al.*, 1996; Starynowicz, 1991, 1993), isonicotinic acid (Chen & Fukuzumi, 2009; Ma, Evans *et al.*, 1999; Wu *et al.*, 2008; Zeng *et al.*, 2000) and isonicotinic acid N-oxide (Mao *et al.*, 1998). In the course of research in this area, our extended group has reported several such compounds with different bridging ligands (Cai *et al.*, 2003; Cui *et al.*, 1999; Zhang *et al.*, 1999). Herein, we report the synthesis and crystal structure of a new lanthanum complex with isonicotinic ligand.

The title compound contains extended [La(C_6_H_4_NO_2_)_2_(H_2_O)_4_]_n_ cationic chains and Cl^-^ anions. The La^III^ ion, lying on a twofold rotation axis, is eight-coordinated by four O atoms belonging to four different isonicotinic ligands [average La—O = 2.451 (3) Å] and four water molecules [average La—O = 2.563 (3) Å] (Fig. 1). The coordination geometry of the La^III^ ion is best described as slightly distorted square-antiprismatic. The La atoms are bridged each other by two *syn*-*syn* µ-*O*:*O*'-carboxylate groups of the isonicotinic ligands, forming an extended chain along [0 0 1]. This geometry is similar to that found in [Eu(*L*)_2_(H_2_O)_4_]_n_.nH_2_O (*L* = isonicotinic acid N-oxide) (Mao *et al.*, 1998) and [La(C_6_H_4_NO_2_)_2_(H_2_O)_4_](NO_3_) (Cai *et al.*, 2003), but differs from those found in Ln(isonicotinate)_3_(H_2_O)_2_ (Ln = Ce, Pr, Nd, Sm, Eu, Tb) (Ma, Evans *et al.*, 1999), in which the Ln^III^ atoms are bridged by four *syn*-*syn* µ-*O*:*O*'-carboxylate groups of the isonicotinic ligands (Ln = Ce, Pr, Nd) or coordinated by both two *syn*-*syn* µ-*O*:*O*'-carboxylate groups and chelating carboxylate groups of the isonicotinic ligands (Ln = Sm, Eu, Tb). To the best of our knowledge, the arrangement in the present complex is rare in the lanthanide analogs.

There are three kinds of hydrogen bonds, O—H···Cl, O—H···O and O—H···N (Table 1). Interchain O—H···N hydrogen bonds between the coordinated water molecules and uncoordinated N atoms of the isonicotinate ligands link the cationic chains into a three-dimensional network with channels along [0 0 1], in which the chloride anions are located, as shown in Fig. 2, forming O—H···Cl hydrogen bonds. Intrachain O—H···O hydrogen bonds are also present. π–π stacking interactions exist between two adjacent isonicotinate ligands located in a same chain [centroid–centroid distance = 3.908 (2) Å].

### Experimental

LaCl_3_.7H_2_O (0.3174 g, 1 mmol), isonicotinic acid (0.2442 g, 2 mmol), NaOH (0.08 g, 2 mmol) were added to a mixture of water (15 ml) and ethanol (10 ml). The resulting mixture was stirred at 423 K for 4 h and filtered off. The filtrate was allowed to stand at room temperature and slow evaporation afforded colorless block crystals of the title complex (yield: 65%). Analysis, calculated for C_12_H_16_ClLaN_2_O_8_: C 29.38, N 5.71, H 3.29%; found: C 29.36, N 5.74, H 3.28%.

### Refinement

H atoms on C atoms were positioned geometrically and refined as riding atoms, with C—H = 0.93 Å and *U*_iso_(H) = 1.2*U*_eq_(C). The water H atoms were located in difference Fourier maps and refined using a riding model, with O—H = 0.85 Å and *U*_iso_(H) = 1.2*U*_eq_(O).

### Crystal data

**Table:**

[La(C_6_H_4_NO_2_)_2_(H_2_O)_4_]Cl	*F*(000) = 960
*M**_r_* = 490.63	*D*_x_ = 1.780 Mg m^−^^3^
Orthorhombic, *P**b**c**n*	Mo *K*α radiation, λ = 0.71073 Å
Hall symbol: -P 2n 2ab	Cell parameters from 3778 reflections
*a* = 8.987 (3) Å	θ = 2.5–28.3°
*b* = 19.769 (3) Å	µ = 2.52 mm^−^^1^
*c* = 10.305 (3) Å	*T* = 296 K
*V* = 1830.8 (9) Å^3^	Block, colorless
*Z* = 4	0.36 × 0.34 × 0.32 mm

### Data collection

**Table:**

Bruker SMART 1000 CCD diffractometer	1652 independent reflections
Radiation source: fine-focus sealed tube	1442 reflections with *I* > 2σ(*I*)
Graphite monochromator	*R*_int_ = 0.029
φ and ω scans	θ_max_ = 25.2°, θ_min_ = 2.1°
Absorption correction: multi-scan (*SADABS*; Sheldrick, 1996)	*h* = −10→10
*T*_min_ = 0.464, *T*_max_ = 0.500	*k* = −13→23
9336 measured reflections	*l* = −12→11

### Refinement

**Table:**

Refinement on *F*^2^	Primary atom site location: structure-invariant direct methods
Least-squares matrix: full	Secondary atom site location: difference Fourier map
*R*[*F*^2^ > 2σ(*F*^2^)] = 0.025	Hydrogen site location: inferred from neighbouring sites
*wR*(*F*^2^) = 0.058	H-atom parameters constrained
*S* = 1.07	*w* = 1/[σ^2^(*F*_o_^2^) + (0.0232*P*)^2^ + 2.6828*P*] where *P* = (*F*_o_^2^ + 2*F*_c_^2^)/3
1652 reflections	(Δ/σ)_max_ = 0.001
110 parameters	Δρ_max_ = 0.46 e Å^−^^3^
0 restraints	Δρ_min_ = −0.83 e Å^−^^3^

### Special details

**Table:**

Geometry. All esds (except the esd in the dihedral angle between two l.s. planes) are estimated using the full covariance matrix. The cell esds are taken into account individually in the estimation of esds in distances, angles and torsion angles; correlations between esds in cell parameters are only used when they are defined by crystal symmetry. An approximate (isotropic) treatment of cell esds is used for estimating esds involving l.s. planes.
Refinement. Refinement of F^2^ against ALL reflections. The weighted R-factor wR and goodness of fit S are based on F^2^, conventional R-factors R are based on F, with F set to zero for negative F^2^. The threshold expression of F^2^ > 2sigma(F^2^) is used only for calculating R-factors(gt) etc. and is not relevant to the choice of reflections for refinement. R-factors based on F^2^ are statistically about twice as large as those based on F, and R- factors based on ALL data will be even larger.

### Fractional atomic coordinates and isotropic or equivalent isotropic displacement parameters (Å^2^)

**Table:**

	*x*	*y*	*z*	*U*_iso_*/*U*_eq_	
O2	0.9557 (3)	0.89408 (12)	−0.0984 (2)	0.0408 (6)	
La1	1.0000	1.011578 (11)	0.2500	0.02190 (10)	
O1	0.8975 (3)	0.92871 (11)	0.1000 (2)	0.0377 (5)	
O3	1.2552 (2)	1.04958 (10)	0.3400 (2)	0.0352 (5)	
H3A	1.2917	1.0890	0.3483	0.042*	
H3B	1.3240	1.0228	0.3163	0.042*	
C1	0.8975 (3)	0.81153 (15)	0.0573 (3)	0.0260 (6)	
C2	0.8242 (4)	0.79557 (17)	0.1717 (3)	0.0374 (8)	
H2	0.7884	0.8293	0.2262	0.045*	
O4	0.7951 (3)	0.95919 (14)	0.3865 (2)	0.0473 (6)	
H4C	0.7803	0.9566	0.4678	0.057*	
H4D	0.7123	0.9541	0.3480	0.057*	
C3	0.9497 (4)	0.75920 (16)	−0.0183 (3)	0.0366 (8)	
H3	1.0001	0.7681	−0.0952	0.044*	
N1	0.8541 (3)	0.67750 (15)	0.1297 (3)	0.0466 (8)	
C5	0.9265 (5)	0.69354 (18)	0.0216 (4)	0.0500 (10)	
H5	0.9633	0.6587	−0.0297	0.060*	
C6	0.8060 (5)	0.7282 (2)	0.2023 (4)	0.0485 (9)	
H6	0.7567	0.7176	0.2790	0.058*	
C7	0.9186 (3)	0.88401 (15)	0.0168 (3)	0.0270 (7)	
Cl1	0.5000	0.94091 (8)	0.2500	0.0543 (4)	

### Atomic displacement parameters (Å^2^)

**Table:**

	*U*^11^	*U*^22^	*U*^33^	*U*^12^	*U*^13^	*U*^23^
O2	0.0554 (15)	0.0312 (12)	0.0358 (13)	−0.0011 (11)	0.0088 (11)	0.0112 (10)
La1	0.02877 (15)	0.01764 (15)	0.01929 (14)	0.000	−0.00134 (9)	0.000
O1	0.0418 (13)	0.0273 (12)	0.0441 (13)	−0.0027 (10)	−0.0035 (11)	−0.0106 (10)
O3	0.0358 (12)	0.0247 (11)	0.0450 (12)	−0.0076 (9)	0.0005 (10)	−0.0071 (10)
C1	0.0297 (16)	0.0252 (16)	0.0232 (14)	−0.0039 (13)	−0.0028 (12)	0.0002 (12)
C2	0.048 (2)	0.0331 (18)	0.0316 (17)	−0.0037 (15)	0.0086 (15)	0.0009 (14)
O4	0.0318 (13)	0.0836 (19)	0.0265 (11)	−0.0182 (12)	−0.0036 (9)	0.0118 (12)
C3	0.050 (2)	0.0280 (17)	0.0322 (17)	−0.0020 (15)	0.0090 (15)	0.0005 (14)
N1	0.0507 (19)	0.0318 (16)	0.057 (2)	−0.0095 (14)	0.0008 (16)	0.0117 (15)
C5	0.067 (3)	0.0247 (18)	0.058 (2)	−0.0007 (19)	0.007 (2)	−0.0048 (17)
C6	0.053 (2)	0.047 (2)	0.045 (2)	−0.0104 (18)	0.0123 (19)	0.0156 (18)
C7	0.0253 (17)	0.0245 (16)	0.0312 (16)	−0.0026 (13)	−0.0032 (13)	0.0012 (13)
Cl1	0.0349 (7)	0.0595 (9)	0.0687 (9)	0.000	−0.0172 (6)	0.000

### Geometric parameters (Å, º)

**Table:**

O2—C7	1.249 (4)	C2—C6	1.379 (5)
La1—O1	2.433 (2)	C2—H2	0.9300
La1—O2^i^	2.465 (2)	O4—H4C	0.8500
La1—O4	2.538 (2)	O4—H4D	0.8500
La1—O3	2.585 (2)	C3—C5	1.378 (5)
O1—C7	1.246 (4)	C3—H3	0.9300
O3—H3A	0.8500	N1—C6	1.323 (5)
O3—H3B	0.8500	N1—C5	1.328 (5)
C1—C3	1.377 (4)	C5—H5	0.9300
C1—C2	1.387 (4)	C6—H6	0.9300
C1—C7	1.504 (4)		
			
C7—O2—La1^ii^	140.0 (2)	O3—La1—O3^iii^	146.21 (10)
O1—La1—O1^iii^	95.35 (11)	C7—O1—La1	149.0 (2)
O1—La1—O2^i^	148.28 (8)	La1—O3—H3A	130.2
O1^iii^—La1—O2^i^	99.69 (8)	La1—O3—H3B	111.2
O1—La1—O2^ii^	99.69 (8)	H3A—O3—H3B	108.5
O1^iii^—La1—O2^ii^	148.28 (8)	C3—C1—C2	118.1 (3)
O2^i^—La1—O2^ii^	81.66 (11)	C3—C1—C7	121.0 (3)
O1—La1—O4	78.60 (8)	C2—C1—C7	120.8 (3)
O1^iii^—La1—O4	69.39 (8)	C6—C2—C1	118.1 (3)
O2^i^—La1—O4	80.82 (8)	C6—C2—H2	121.0
O2^ii^—La1—O4	141.00 (8)	C1—C2—H2	121.0
O1—La1—O4^iii^	69.39 (8)	La1—O4—H4C	133.2
O1^iii^—La1—O4^iii^	78.60 (8)	La1—O4—H4D	115.2
O2^i^—La1—O4^iii^	141.00 (8)	H4C—O4—H4D	108.4
O2^ii^—La1—O4^iii^	80.82 (8)	C1—C3—C5	119.2 (3)
O4—La1—O4^iii^	131.82 (13)	C1—C3—H3	120.4
O1—La1—O3	139.41 (7)	C5—C3—H3	120.4
O1^iii^—La1—O3	68.40 (7)	C6—N1—C5	117.0 (3)
O2^i^—La1—O3	72.31 (8)	N1—C5—C3	123.4 (3)
O2^ii^—La1—O3	82.19 (8)	N1—C5—H5	118.3
O4—La1—O3	124.28 (7)	C3—C5—H5	118.3
O4^iii^—La1—O3	70.97 (7)	N1—C6—C2	124.3 (3)
O1—La1—O3^iii^	68.40 (7)	N1—C6—H6	117.8
O1^iii^—La1—O3^iii^	139.41 (7)	C2—C6—H6	117.8
O2^i^—La1—O3^iii^	82.19 (8)	O1—C7—O2	125.6 (3)
O2^ii^—La1—O3^iii^	72.31 (8)	O1—C7—C1	117.7 (3)
O4—La1—O3^iii^	70.97 (7)	O2—C7—C1	116.7 (3)
O4^iii^—La1—O3^iii^	124.28 (7)		

Symmetry codes: (i) *x*, −*y*+2, *z*+1/2; (ii) −*x*+2, −*y*+2, −*z*; (iii) −*x*+2, *y*, −*z*+1/2.

### Hydrogen-bond geometry (Å, º)

**Table:**

*D*—H···*A*	*D*—H	H···*A*	*D*···*A*	*D*—H···*A*
O3—H3*A*···N1^iv^	0.85	1.85	2.699 (4)	175
O3—H3*B*···Cl1^v^	0.85	2.36	3.212 (2)	175
O4—H4*C*···O3^vi^	0.85	2.01	2.860 (3)	180
O4—H4*D*···Cl1	0.85	2.17	3.024 (3)	180

Symmetry codes: (iv) *x*+1/2, *y*+1/2, −*z*+1/2; (v) *x*+1, *y*, *z*; (vi) −*x*+2, −*y*+2, −*z*+1.
